# Supplementary material for: Communication Disparities between Nursing Home Team Members
Source: Int J Environ Res Public Health. 2022 May 14;19(10):5975. doi: 10.3390/ijerph19105975 (PMC9141434; doi:10.3390/ijerph19105975)
Supplement: Supplementary file 1 [file ijerph-19-05975-s001.zip › ijerph-1687620-supplementary.pdf]

International Journal of
Environmental Research and Public Health

Supplemental Content

Long-Term Care Communication Survey Question 1

This anonymous survey asks for your responses to questions about communication in long-term care facilities. The questions address both your communication with providers and staff in long-term care teams as well as your communication with residents and their families. The survey results will help design educational offerings to improve communication in long-term care facilities. We thank you in advance for your time and effort in completing this survey. The survey should take no more than about 10 min.

1. When I communicate my concerns about the resident to those listed below, my input is:

	Not Valued	Somewhat Valued	Mostly Valued	Highly Valued	Not Applicable
Activities Director	1	2	3	4	NA
Administrators	1	2	3	4	NA
Certified Nursing Assistants	1	2	3	4	NA
Dietary	1	2	3	4	NA
Director of Nursing	1	2	3	4	NA
Front Desk / Office	1	2	3	4	NA
Housekeeping/Laundry	1	2	3	4	NA
Human Resources	1	2	3	4	NA
Licensed Nursing / MDS Coordinator	1	2	3	4	NA
Maintenance	1	2	3	4	NA
Medical Technicians	1	2	3	4	NA
Nurses	1	2	3	4	NA
Occupational Therapy	1	2	3	4	NA
Other members of my discipline	1	2	3	4	NA
Pharmacists	1	2	3	4	NA
Physical Therapy	1	2	3	4	NA
Physicians	1	2	3	4	NA
Social Workers	1	2	3	4	NA
Speech Therapy	1	2	3	4	NA
Other: (Please Specify)	1	2	3	4	NA
